# SNPs-Panel Polymorphism Variations in *GHRL* and *GHSR* Genes Are Not Associated with Prostate Cancer

**DOI:** 10.3390/biomedicines11123276

**Published:** 2023-12-11

**Authors:** Nesrine Merabet, Nicolas Ramoz, Amel Boulmaiz, Asma Bourefis, Maroua Benabdelkrim, Omar Djeffal, Emmanuel Moyse, Virginie Tolle, Hajira Berredjem

**Affiliations:** 1 Laboratory of Applied Biochemistry and Microbiology, Department of Biochemistry, Faculty of Sciences, Badji Mokhtar University, Annaba 23000, Algeria; boulmaiz.a@gmail.com (A.B.); asma_bourefis@yahoo.com (A.B.); maroua_benabdelkrim@yahoo.fr (M.B.); 2 Unit 85 PRC (Physiology of Reproduction and Behavior), Centre INRAe of Tours, University of Tours, 37380 Nouzilly, France; emmanuel.moyse@univ-tours.fr; 3 University Paris Cité, INSERM U1266, Institute of Psychiatry and Neuroscience of Paris (IPNP), 75014 Paris, France; nicolas.ramoz@inserm.fr (N.R.); virginie.tolle@inserm.fr (V.T.); 4 Private Medical Uro-Chirurgical Cabinet, Cité SafSaf, BatR02 n°S01, Annaba 23000, Algeria; omar_djeffal@yahoo.fr

**Keywords:** prostate cancer, *GHRL*, *GHSR*, genetic polymorphisms, SNPs, MDR

## Abstract

Prostate cancer (PCa) is a major public health problem worldwide. Recent studies have suggested that ghrelin and its receptor could be involved in the susceptibility to several cancers such as PCa, leading to their use as an important predictive way for the clinical progression and prognosis of cancer. However, conflicting results of single nucleotide polymorphisms (SNPs) with ghrelin (*GHRL*) and its receptor (*GHSR*) genes were demonstrated in different studies. Thus, the present case–control study was undertaken to investigate the association of *GHRL* and *GHSR* polymorphisms with the susceptibility to sporadic PCa. A cohort of 120 PCa patients and 95 healthy subjects were enrolled in this study. Genotyping of six SNPs was performed: three tag SNPs in *GHRL* (rs696217, rs4684677, rs3491141) and three tag SNPs in the *GHSR* (rs2922126, rs572169, rs2948694) using TaqMan. The allele and genotype distribution, as well as haplotypes frequencies and linked disequilibrium (LD), were established. Multifactor dimensionality reduction (MDR) analysis was used to study gene–gene interactions between the six SNPs. Our results showed no significant association of the target polymorphisms with PCa (*p* > 0.05). Nevertheless, SNPs are often just markers that help identify or delimit specific genomic regions that may harbour functional variants rather than the variants causing the disease. Furthermore, we found that one *GHSR* rs2922126, namely the TT genotype, was significantly more frequent in PCa patients than in controls (*p* = 0.040). These data suggest that this genotype could be a PCa susceptibility genotype. MDR analyses revealed that the rs2922126 and rs572169 combination was the best model, with 81.08% accuracy (*p* = 0.0001) for predicting susceptibility to PCa. The results also showed a precision of 98.1% (*p* < 0.0001) and a PR-AUC of 1.00. Our findings provide new insights into the influence of *GHRL* and *GHSR* polymorphisms and significant evidence for gene–gene interactions in PCa susceptibility, and they may guide clinical decision-making to prevent overtreatment and enhance patients’ quality of life.

## 1. Introduction

Prostate cancer (PCa) is the most common solid tumour after lung cancer and the second most common cause of cancer death in men [1]. Measurement of prostate serum antigen (PSA) levels remains the current standard for diagnosing PCa [2]. Early diagnosis of PCa is critical for the early management of patients with this disease. Unfortunately, PSA has significant limitations in that non-tumour conditions such as infections and inflammation can also increase PSA levels [3]. As a result, many unnecessary biopsies are performed, leading to adverse patient outcomes and increased public costs. Hence, considerable effort has been devoted trying to identify risk factors that could complement or even replace plasma PSA to improve the diagnosis of PCa [4,5].

Ghrelin is a brain–gut peptide with 28 amino acids that was isolated as the endogenous ligand for the ghrelin receptor. The preproghrelin gene is located on chromosome 3q26.31 [6]. Ghrelin was discovered as a peptidic growth-hormone-releasing and appetite-stimulating hormone that is secreted by the stomach mucosa [7,8]. The ghrelin signalling system comprises a pleiotropic and complex network of several peptides, including native ghrelin [9], and receptors (GHSR1a/b), involved in the regulation of multiple pathophysiological processes [10]. Ghrelin has two major forms: the acylated form (n-octanoylated) and unacylated (nonoctanoylated) form. Octanoylation of ghrelin is critical for its physiological functions which depend upon ghrelin O-acyltransferase (GOAT) catalyzation. Ghrelin receptors are two splice variants GHSR1a and GHSR1b, which are G protein-coupled and widely expressed. GHSR1a is considered as the main functional receptor that mediates most of the physiologic effects. GHSR1b is a truncated splice variant form of GHSR1a [11,12].

Since the first publication reporting the antiproliferative effect of ghrelin, numerous cancer studies suggested a role for the ghrelin/GHSR system in various tumours, modulating proliferation, apoptosis, and metastasis [13,14,15]. However, several other studies have shown that ghrelin has proliferative effects on various cancer cell lines [16,17,18]. The differential expression of ghrelin receptors as a potential explanation for the controversial role of ghrelin in different cancers was highlighted by Gahete et al. [9]. Human prostate carcinomas and benign neoplasms express ghrelin and GHSR mRNA. However, in normal prostate tissue, ghrelin mRNA expression is undetectable, suggesting that ghrelin is involved in the pathophysiology of PCa [15,19].

Gene polymorphism describes individual variations in alleles or genotypes at the same locus in the population, which can change the expression of a gene and influence the occurrence of disease or increase/decrease the population’s susceptibility to some diseases [20]. Several polymorphisms of ghrelin and its receptor have been described, particularly in diabetes [21,22], obesity [23], metabolic disorders [24,25], cardiovascular disease [26], hepatitis [27], anorexia-cachexia [28,29], and alcoholism [30,31]. The ghrelin polymorphisms most studied to date are located in the promoter and coding regions of the gene; some of them have implications for gene activity [21]. The most studied missense SNPs in the pre-proghrelin gene (*GHRL*) to date are rs34911341/Arg51Gln, rs4684677/Gln90Leu, and rs696217/Leu72Met. Among these SNPs, studies have provided evidence for the role of the rs4684677 T/A polymorphism in influencing metabolic syndrome, panic disorder, cancer, and autoimmune thyroid disease [32,33,34,35].

To date, advances in our understanding of the association of allelic variants of ghrelin and its receptor with cancer have been directed primarily towards oesophageal cancer [36], colorectal cancer [37,38], breast cancer [39], and non-Hodgkin’s lymphoma [40]. These studies suggested by analogy that *GHRL* and *GHSR* polymorphisms could have a role in PCa. However, very few studies have examined the association of genes with PCa risk [34]. Thus, some studies examined the association of growth hormone (GH) and ghrelin gene polymorphisms with PCa risk and found significant associations between certain GH, ghrelin SNPs polymorphisms and PCa risk. This demonstrated that the ghrelin risk SNP rs4713266 is associated with an increased risk of PCa in African patients [41,42]. On the other hand, the presence of the In1-ghrelin splice variant has been observed in PCa tissues and was associated with increased aggressiveness characteristics of prostate cancer cells [43,44]. Further research is therefore needed to confirm these associations and explore the potential mechanisms underlying the relationship between ghrelin/GHSR polymorphisms and PCa risk.

This is the first comprehensive analysis of six functional polymorphisms of *GHRL* (rs696217, rs4684677, and rs34911341) and *GHSR* (rs2922126, rs572169, and rs2948694). genes. To achieve this objective, we investigated the possible association between these SNPs and sporadic PCa among Algerian subjects, with respect to clinicopathological features and in an adequate follow-up interval and frequency in accordance with each PCa patient’s susceptibility to progression and death. Overall, survival was assessed by Kaplan–Meier curves. Moreover, multifactor dimensionality reduction (MDR) analysis was used to study gene–gene interactions between the six SNPs and PCa risk.

## 2. Materials and Methods

### 2.1. Study Population

The local Ethics Committee of the University of Badji Mokhtar, Annaba, approved this study under the number CEDUBMA-02-12/21, which was conducted in accordance with the Declaration of Helsinki. Prior to enrollment, all patients and control individuals gave written informed approval. To ensure privacy and confidentiality, subjects were given a confidential identification number.

This prospective case–control study was conducted during the period 2017–2019 and involved 215 individuals. The patient group consisted of 120 individuals with histologically confirmed sporadic PCa diagnosed at different grades of severity; patients were aged between 51 and 94 years (71.59 ± 8.476). All clinico-pathological information was collected from the clinical records by the attending clinician and summarized in Table 1. The control group consisted of 95 healthy volunteers aged between 29 and 82 years (74.63 ± 9.50). The incidence of death in the patient group was estimated at 15.43%. The incidence of PCa was higher in patients over 65 than in those under 65 years. The most frequent Gleason score (GS) was 7, accounting for 40% of cases, compared to GS < 7 (31.66%) and GS > 7 (28.33%). The clinical stage was predominantly localised (58.33%) compared with advanced (28.33%) and metastatic (13.33%). There was a statistically significant difference in the distribution of age and PSA between patients and controls (*p* < 0.05).

The diagnosis of PCa was established according to serum PSA levels ≥ 4.0 ng/mL, the presence of a palpable nodule on a digital rectal examination of the prostate, and the anatomo-pathological characteristics. Patients were followed for up to five years. There was no family history of PCa in any of the participants. There was a statistically significant difference in the body mass index (BMI) between the healthy individuals and the PCa patients (*p* = 0.013).

Exclusion criteria for patients included any previous history of PCa. Controls and patients also had to be free of all other forms of cancer.

### 2.2. DNA Extraction

Genomic leukocyte DNA was extracted from 10 mL of whole blood collected on EDTA using the FlexiGene^®^ DNA kit (Qiagen, Hilden, Germany) according to the manufacturer’s recommendations. The DNA concentration in each sample was measured using a NanoDrop™1000 spectrophotometer (Thermo Fisher Scientific, Waltham, MA, USA). Finally, the genomic DNA was dissolved in TE 10/1 buffer (10 mM Tris/HCl; 1 mM EDTA; pH = 8.0) at the concentration of 10 ng/µL and stored at −20 °C.

### 2.3. Genotyping

A total of 215 participants were genotyped. Genetic polymorphisms of 3 *GHRL* and 3 *GHSR* allelic variants were analyzed by TaqMan^®^ probe assays (Table 2).

PCR reactions were performed in a 10 µL reaction mixture containing 10 ng of genomic DNA, 1× PCR buffer, 3 mM MgCl_2_, 200 μM of each dNTP, 200 nM of each primer and probe, and one unit (U) of Taq DNA polymerase (Invitrogen, Thermo Fisher Scientific Inc., Strasbourg, France). For each amplification run, a negative control containing no DNA was performed to check for contamination. The amplification was carried out in the SDS (Sequence Detection System) 7900HT Fast Real-Time PCR System (Applied Biosystems, Thermo Fisher Scientific Inc., Strasbourg, France). The amplification program consisted sequentially of an initial denaturation step at 95 °C for 10 min, followed by 40 cycles, each comprising denaturation at 92 °C for 15 s and a hybridization/elongation step at 60 °C for 60 s. Genotype calls were assigned according to the sample position on the allelic discrimination plot.

### 2.4. Statistical Analysis

All data were analyzed using GraphPad 8 Software Inc., LA Jolla, CA, USA. Comparisons between the two populations were performed, in the form of the mean ± SD, using Pearson’s Chi-square test. The association between ghrelin (*GHRL*) polymorphims, ghrelin receptor (*GHSR*) polymorphisms, and PCa was analyzed using multivariate logistic regression. Odds ratios (ORs) were used to estimate genetic associations with PCa with a 95% confidence interval (CI). The Hardy–Weinberg equilibrium (HWE) values were checked. Results were considered significant when the probability *p*-value was less than 0.05. The Fisher correction was applied when the sample size was less than 3.

To calculate genotypic, allelic, haplotypes frequencies and LD, we used Haploview 4.1 software [45] available free of charge at the following address: https://www.broadinstitute.org/haploview/haploview (accessed on 7 June 2022). Haplotypes were estimated using an accelerated expectation-maximization algorithm similar to the partition/ligation method to create highly accurate population frequency estimates of the phased haplotypes.

Our recruitment of sample size provides an enrolment ratio of 0.79, and an estimated genetic statistical power of 80% is computed for a difference of 20% of allelic frequencies between controls and patients.

Survival analysis was performed using the Kaplan–Meier curve. Multifactorial dimensionality reduction (MDR) analysis was performed using MDR v3.0.2 statistical software available free of charge at http://sourceforge.net/projects/mdr (accessed on 26 April 2023), Computational Genetics Laboratory, University of Pennsylvania, PA, USA) based on the algorithm of Ritchie et al. [46] and Hahn et al. [47]. The MDR method was designed to predict gene–gene interactive effects (epistasis) in datasets containing categorical independent variables SNPs. This method can be summarized in 3 main steps: (i) calculation of the case/control ratio for each combination of genotypes, (ii) classification of each combination as ‘high risk’ or ‘low risk’, (iii) estimation of the prediction error via cross-validation [46,48].

## 3. Results

### 3.1. Association of GHRL and GHSR SNPs with PCa Risk

A total of six SNPs were successfully genotyped in PCa cases and controls. For the studied polymorphisms, we did not observe any deviation from HWE in the control group. However, it should be noted that the SNPs rs696217 (*GHRL*) and rs2922126 rs572169 (*GHSR*) were not in HWE for PCa patients (Table 3). A deviation from HWE may be the consequence of a genotyping bias leading to an excess of heterozygotes.

The genotyping success rate was over 90%. Detailed information on genotypes and alleles is presented in Table 4 and Table 5. The distribution of alleles at *GHRL* and *GHSR* genes was identical between the control and patient groups. In the control population, the allelic distribution revealed that for the SNPs rs696217, rs4684677, rs2922126, rs572169, and rs2948694, the major alleles were G (82.1%), A (99.4%), A (67.8%), C (73.8%), and A (98.2%) respectively. In patients, the frequencies of the major alleles for these same SNPs were: G (78.5%), A (99.5%), A (62.5%), C (72.1%), and A (96.2%), respectively (Table 4).

Statistical analysis showed no significant difference in the distribution of allelic frequencies between patients and controls (*p* > 0.05).

Genotyping analysis enabled us to distinguish homozygous from heterozygous genotypes for each explored SNP. Table 5 shows the genotype distribution of PCa patients and healthy controls. No statistically significant association of the studied SNPs with PCa was demonstrated (*p* > 0.05), except for the T/T genotype of rs2922126, the *GHSR* gene which was significantly more frequent in patients than in controls (*p* = 0.040). These data suggest that this genotype could be a PCa susceptibility genotype.

**Figure 1:**
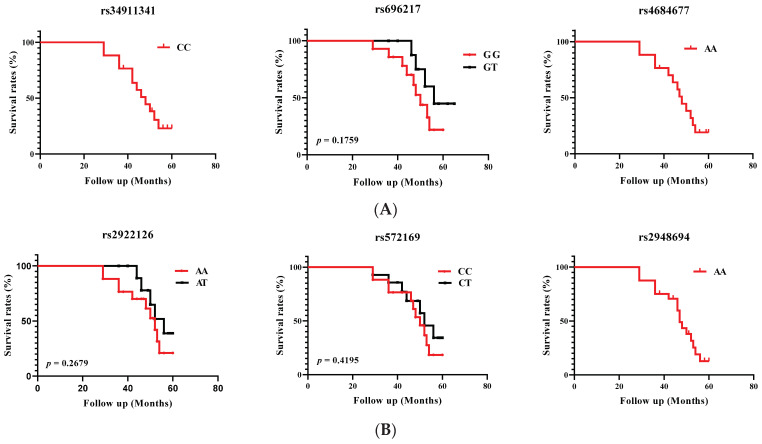
Overall survival curves for the occurrence of death in PCa patients according to (**A**) *GHRL* and (**B**) *GHSR* genotypes. Survival was calculated over 60 months.

Survival associations were assessed using the Kaplan–Meier method. The time to an event (death) was measured as the time between diagnosis and the end of the event, approximately 5 years (60 months after diagnosis) or the last observation period. Patient groups were defined by the genotypes of the six studied SNPs. Patients alive at the last follow-up were censored, deaths being considered as an event. The overall results are shown in Figure 1.

We were unable to compare survival curves using the Kaplan–Meier method or to define probability using the log rank test for rs34911341 (CC genotype), rs4684677 (AA genotype), and rs2948694 (AA genotypes) variants, as there were no heterozygous patients. Kaplan–Meier survival analysis showed a non-significant (*p* > 0.05) decrease in overall survival associated with *GHRL* (rs696217) and *GHSR* (rs2922126 and rs572169) variants.

### 3.2. Analysis of GHRL and GHSR Haplotypes with PCa Risk

We performed an analysis of the most likely haplotype blocks with Haploview software to determine a possible association with an increased risk of PCa. Table 6 shows the analysis of haplotypes according to the physical order of the gene markers rs34911341, rs696217, and rs4684677 (*GHLR*) and rs2922126, rs572169, and rs2948694 (*GHSR*). This made it possible to define two haplotypes for the *GHRL* gene, with frequencies ranging from 19.7% to 79.8%, and six haplotypes for the *GHSR* gene, with frequencies ranging from 0.015% to 58.7%.

The frequencies of the different haplotypes of the studied genes, in both patients and controls, were very similar; the differences were not statistically significant (*p* > 0.05). According to the obtained results, the most frequent haplotype for *GHRL* and *GHSR* was AGC (79.8%) and ACA (58.7%), respectively.

### 3.3. Linkage Disequilibrium Analysis of the GHRL and GHSR SNPs

**Figure 2:**
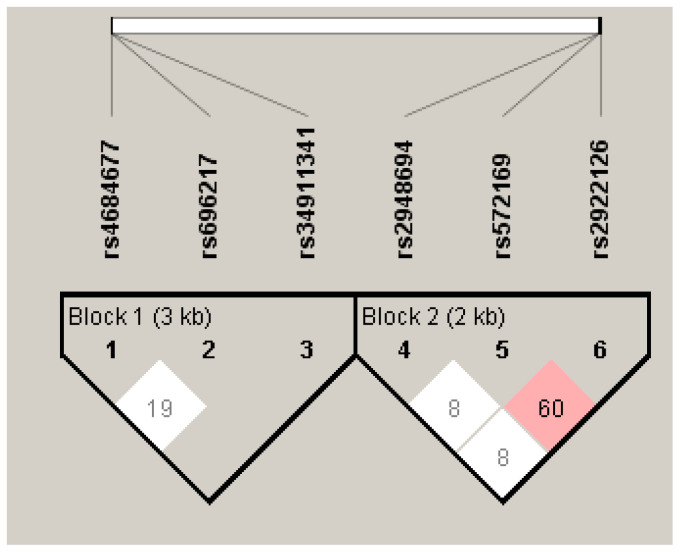
Degree of LD of *GHRL* (Block 1) and *GHSR* (Block 2) SNPs.

Linkage disequilibrium (LD) analysis showed that for the *GHRL* gene, only the combination of SNPs rs696217 and rs4684677 was in a low LD (D′ = 0.190) (Figure 2). The combinations rs696217 and rs34911341, as well as rs4684677 and rs34911341, were not in LD. However, it is difficult to consider these LDs with any relevance given the small number of patients carrying the AG genotype for the rs4684677 variant.

For the *GHSR* gene, the rs2922126 and rs572169 combination has an average LD (D′ = 0.600) (Figure 2). Hence, the probability of two mutated alleles being transmitted simultaneously is high, which makes it difficult to attribute the observed associations to a given polymorphism. The SNPs combinations rs2922126 and rs2948694, as well as rs572169 and rs2948694, are not in LD (D′ = 0.08).

### 3.4. Multifactor Dimensionality Reduction (MDR)

The MDR is a non-parametric model selection method. It was designed to predict gene–gene (epistasis) or gene–environment interactive effects in datasets with categorical independent variables such as SNPs. The concept is to consider, one by one, all possible combinations of loci. It involves performing a 10-fold cross-validation on the dataset divided into ten equal parts, with nine subclasses serving as the base training set for the cross-validation and one as the test. The process is repeated ten times and the final candidate gene–gene interaction model is selected on the basis of maximum cross-validation consistency (CVC) and maximum balanced test accuracy. In addition, this method starts with a structure with several markers and several genotypes at the markers (high-dimensional structure) and ends with a high-risk genotype/low-risk genotype structure (one-dimensional structure). Each combination of loci results in a model which classifies individuals with a low-risk genotype as non-diseased and a high-risk genotype as diseased. The model that maximises the case–control ratio of high-risk genotypes is selected from all the models of the same size. The models of each size are evaluated by four measures: the associated trainer accuracy, the associated testing accuracy, and the CV consistency. These measures allow us to make a decision about the presence and nature of genetic interactions suggested by the data [46,48]. Interaction entropy plots were constructed to interpret the combination effects identified by the MDR.

**Figure 3:**
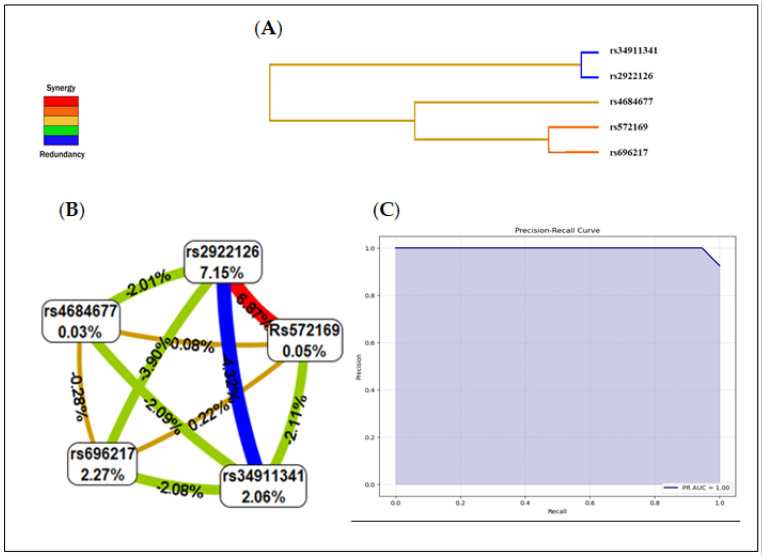
Summary of interaction models using MDR. (**A**) Dendrogram of the interactions between the studied SNPs. (**B**) Interaction map for PCa risk. (**C**) Precision recall receiver operating characteristic (PR−ROC) curve depicting the clinical interest of this model: The PR−AUC was 1.00 with a 91.6% accuracy in predicting PCa risk.

SNPs from the *GHRL* and *GHSR* genes were included in the gene–gene interaction model. Table 7 summarizes the results of the exhaustive MDR analysis evaluating all possible combinations of the six SNPs included to predict the interaction models. The presence of the rs2922126 and rs572169 polymorphisms represented the best model among all the factors included. This model had a maximum balanced test precision of 0.8108, a CVC of 10/10, and a significant *p*-value of 0.0001. The interactions between *GHRL* and *GHSR* gene polymorphisms in PCa patients are represented at the dendrogram level (Figure 3A). The colours represent the degree of synergy, ranging from red (highest information gain) to blue (information redundancy).

The interaction map of all polymorphisms assessed for PCa risk based on entropy measures between individual variables revealed a strong synergistic interaction effect between rs2922126 and rs572169 with an information gain of 6.87% (red line). The values within the nodes indicate the information gain of the individual attributes or main effects, while the values between the nodes indicate the information gain of the pairwise combinations of attributes or interaction effects.

Hence, the individual contributions of the polymorphisms rs696217 (2.27%), rs4684677 (0.03%), rs34911341 (2.06%), rs2922126 (7.15%), and rs572169 (0.05%) presented in Figure 3B correlate well with the non-significant associations described in Table 3; moreover, the interaction between these same SNPs was redundant or antagonistic (blue and green lines). The interaction between rs696217 and rs4684677 on the one hand, and rs2922126 and rs572169 on the other correlates perfectly with the LD results.

As shown in Figure 3C, the MDR model revealed a risk-prediction model which had a 91.6% accuracy in predicting PCa risk with a precision of 98.1% (*p* < 0.0001). The area under the ROC curve PR-AUC was 1.00.

The F1-score is a combined measure of precision and sensitivity. It is calculated according to the formula: F1 = 2 × (Precision × Sensitivity/Precision + Sensitivity). The F-value was 0.953, and the Matthews correlation coefficient (MCC) was 1.544.

The attained PR−AUC value of 1 in our model signifies an exemplary performance, achieving maximal precision and recall across all classification thresholds. This exceptional result underscores the model’s robust ability to accurately identify positive instances while minimizing false positives, demonstrating its efficacy in handling the complexities of the given task with unparalleled precision and recall.

## 4. Discussion

It is currently accepted that certain metabolic alterations such as diabetes and obesity are closely linked to an increased risk of cancer and cancer-related mortality [49,50]. Thus, several endocrine/metabolic factors such as certain components of the ghrelin system, have been associated with PCa [19,51].

Polymorphisms in the ghrelin gene and ghrelin receptor gene have been linked to a variety of outcomes in cancer, including increased risk, protection from cancer, and no association. This study highlighted an anomaly in the distribution of allelic variants of ghrelin (rs696217) and its receptor (rs2922126 and rs572169) in patients with PCa; the respective levels of these alleles differ from HWE. This would be due to a low rate of heterozygotes among the studied individuals. In addition, no significant association was observed between the *GHRL*/*GHSR* polymorphisms and PCa, although a better response seems to emerge for TT homozygotes of the rs2922126 (*GHSR*) variant (*p* = 0.040). In this study, the ghrelin frequency of homozygotes was higher than that of heterozygotes, in contrast to the ghrelin receptor for which the frequency of heterozygotes was higher than that of homozygotes for both variants rs2922126 and rs572169. The AG genotype for variants rs4684677 and rs2948694 showed a very marked difference, with frequencies of 0.009 and 0.076, respectively. Our study also showed that the heterozygous CT genotype for the *GHRL* rs34911341 polymorphism was absent in both controls and patients. It seems to be a very rare genotype. This result corroborates that found by Zhang et al. [52] who showed that among a cohort of 600 subjects, only one subject had the CT genotype for the *GHRL* rs34911341 polymorphism. A meta-analysis of case–control studies showed that the rs4684677 polymorphism in the *GHRL* gene and the rs572169 polymorphism in the *GHSR* gene confer an increased risk of breast cancer, whereas the rs696217 and rs2075356 polymorphisms in the *GHRL* gene protect carriers against breast cancer [34]. Furthermore, in another case–control study, two SNPs in the *GHRL* ghrelin gene, namely rs27647 and rs35683, were associated with a lower risk of developing colorectal cancer [37]. These results suggest that genetic variations in the ghrelin gene may play a role in the development of cancer. However, there is very little information on the association between ghrelin polymorphisms and PCa risk.

This study, based on the exploration of six SNPs, is the first to provide new evidence that the ghrelin gene and its receptor are not associated with PCa and indicates that cancer cells’ proliferation is not directly increased by ghrelin. By promoting the PI3K/AKT and MAPK pathways [53] and sustaining resistance to apoptosis [54], ghrelin stimulates proliferation in normal cells. Variations in the amount of *GHSR* expression may contribute to the disparities in cell proliferation and resistance to apoptosis between cancer and normal cells.

The overall survival curves for the occurrence of death in PCa patients according to genotype showed a non-significant (*p* > 0.05) decrease in overall survival associated with the *GHRL* (rs696217) and *GHSR* (rs2922126 and rs572169) variants. A remarkable fact is that homozygous individuals showed a low survival rate, unlike heterozygous individuals which could be linked to better survival. Carriers of the T allele appeared to have better patient survival. In contrast, haplotypes of the *GHSR* gene did not seem to have any effect on PCa. Associations with alleles can be the result of chance, can indicate that an allele is functional, can be a risk factor, or can be the result of an allele being in LD with an unknown locus that affects the phenotype [55].

To our knowledge, there are no studies examining the effect of the haplotypes and LD of the studied SNPs on PCa risk. Several studies suggest that haplotype analysis is more informative than the analysis of isolated polymorphisms [56]. In fact, the use of haplotypes (alleles combined on the same chromosome) instead of genotypes (isolated polymorphisms) is generally considered to be a major advance as it allows better interpretation in association analyses. Furthermore, the LD is used to predict the simultaneous evolution of two loci located on the same chromosome or haplotype. It is determined by the physical distance between markers. The greater the LD, the greater the probability that the two alleles will be transmitted together, which is particularly the case for two polymorphisms that are physically close on the chromosome. Thus, in the case of a high positive LD, the probability of two mutated alleles being transmitted simultaneously is high, making it difficult to attribute the observed associations to a given polymorphism [57,58]. In principle, if the underlying polymorphism structures were known, it would be possible to considerably reduce the number of SNPs to be used for association studies.

Although we defined two haplotypes for the *GHRL* gene and six haplotypes for the *GHSR* gene, no statistically significant difference was observed (*p* > 0.05). Moreover, the combination of rs2922126 and rs572169 for the *GHSR* gene shows an average LD (D′ = 0.600), suggesting that these loci jointly may affect the gene polymorphisms. Functional variants could be located in the *GHSR* gene or in other genes around these loci, independently or synergistically, and exert an increased risk of PCa. Hence, the probability of two mutated alleles being transmitted simultaneously is high; it becomes difficult to link the observed association to a specific polymorphism. Thus, the selection of an informative subset of common SNPs for use in association studies is necessary to obtain sufficient power to assess the causal role of common DNA variations in complex disease situations. Studies with larger sample sizes and different populations are needed to gain detailed insight into the link between genetic variations in this region and PCa. Otherwise, although we did not investigate the effects of mutations on *GHRL*/*GHSR* gene expression, several studies have reported the impact of mutations on gene expression [59,60].

The association between ghrelin polymorphisms and PCa has been a matter of interest in recent years. However, little is known about the physiopathological mechanisms. Growing data suggest that ghrelin regulates several processes linked to the expansion of cancer; however, because of systemic variability and experimental methods, the exact function of ghrelin remains unknown [61]. An association study does not provide enough information to determine whether the observed allele is functionally responsible for the effect. Additional investigation into the pleiotropic effect of the alleles may yield insights, but *in vitro* and *in vivo* functional analyses are necessary to definitively determine the impact of the genotyped alleles [62].

Although it does not properly rule out *GHRL* and *GHSR* as potential PCa candidate biomarker genes, we did not find any evidence in our cohort that the studied gene variants are associated with PCa. This may indicate that the actual *GHRL* and *GHSR* etiological variants involved in the pathogenesis of PCa are still unknown. We might take into account the possibility that additional gene polymorphisms contribute to PCa susceptibility. Moreover, their role in the pathology of PCa, if any, would likely involve different molecular events. No data are currently available for these polymorphic alleles. Therefore, more research in this field is required.

When we evaluated the polymorphisms individually, we observed that the effect is relatively small to be detected as statistically significant with our sample size. However, when the polymorphisms are analysed in combination using the multifactorial dimensionality reduction (MDR) method, a significant interaction can be identified since the combined effect of the polymorphisms is stronger. One of the advantages of the MDR method is that it does not require large sample sizes to detect significant interactions [63]. On the other hand, its interest lies in the fact that disease risk depends on the particular combination of inherited genotypes. The MDR method was the first innovative tool generated mostly to detect and categorize non-additive genetic interactions in population-based investigations of human disease. The initial version of the MDR approach was developed in 2001 by Ritchie et al. to identify SNPs interactions correlated with data related to breast cancer [46,64].

Otherwise, identifying the interactions between multiple genetic variants is a major challenge in elucidating the aetiology of common diseases such as cancer, which is thought to be multifactorial, caused by genetic variants at several loci, each locus conferring a modest risk of developing the disease [46,65,66]. Multiple and complex interactions underlie gene expression and regulation, and there is evidence that gene–gene interactions play a role in determining the phenotypes of common diseases [67,68]. The most common type of genetic variation is the single nucleotide polymorphism. SNP data are often analysed using single locus methods [69,70]. Loci that interact in complex ways may not be easily detected using such methods [71]. In this context, the MDR method was designed to detect associations between several genetic markers and a phenotype by examining higher-order interactions between SNPs in a case–control situation [46,47,72]. MDR searches a large volume of SNPs data in order to identify a combination of cancer-related attributes by reducing the number of misclassified individuals. It combines two or more variables into a single variable (resulting in a reduction in dimensionality); this changes the data representation space and facilitates the detection of non-linear interactions between variables [65,73]. Combinatorial methods examine all possible combinations of loci in order to identify combinations of SNPs that are predictive of a discrete clinical parameter. A particular combination of SNPs, when combined with the correct non-linear function, is a significant predictor of disease susceptibility.

In this study, we performed for the first time an MDR data-mining approach to detect the gene–gene interactions of six SNPs of *GHRL*/*GHSR* genes in PCa. To our knowledge, this is the first report on the interaction effect of *GHRL* and *GHSR* polymorphism in PCa patients analysed by MDR. It is a data-mining and machine-learning approach that involves non-parametric model-free methods for estimating non-linear interactions with few false positives, even on relatively small samples. The aim is therefore to detect and characterize non-linear (epistatic) interactions between DNA sequence variations in human populations. Understanding the role of DNA sequences in disease susceptibility is therefore capable of improving diagnosis, prevention, and treatment. Validation of the models by permutation tests and false positive ratio probabilities were also carried out to overcome inaccurate estimation.

There are two types of epistasis, biological and statistical. Biological epistasis results from physical interactions between biomolecules (DNA, RNA, proteins, enzymes, etc…) and occurs at the cellular level in an individual. Statistical epistasis, on the other hand, occurs at the population level and is achieved when there is inter-individual variation in DNA sequences [74]. Our study focuses on the detection and characterization of statistical epistasis in a population of PCa patients, using MDR, which was developed specifically for this field. The MDR analysis showed an epistatic interaction between the SNPs rs572169 and rs2922126. This combination in PCa susceptibility highlighted the best model that included values of learning equilibrium accuracy and testing equilibrium accuracy and the highest CVC. Thus, our study revealed a significant interaction (*p* = 0.0107) between the polymorphisms rs572169 and rs2922126 with a CVC of 10 and an entropy value of 6.87%. Moreover, the F-value was 0.953, the Matthews correlation coefficient (MCC) was 1.544, and the area under the ROC curve PR-AUC was 1.00.

The MDR two-loci model was therefore the best predictor of risk in PCa patients. High- and low-risk genotypes were identified between these two polymorphisms. This combined model confers a risk of PCa, but individually, these SNPs have no effect. In gene–gene interaction, the strength of the link is based on entropy levels. A positive percentage of entropy denotes a synergistic interaction, while a negative percentage denotes redundancy. In the case of the gene–gene interaction in our study, the order of strength of association of the variables is as follows: rs2922126 > rs696217 > rs34911341 > rs572169 > rs4684677. A synergistic interaction (entropy: 6.87%) was observed between rs2922126 and rs572169. Strong redundancy was observed between rs2922126 and rs34911341. Individually, these SNPs showed no effect.

In the literature, there are no MDR studies on PCa. The MDR method has been successfully applied to the detection of epistasis or gene–gene interactions in a variety of complex human diseases, including some cancers with different locations. In a literature review [64], several works on the application of MDR for the analysis of gene–gene interactions are cited. In order to identify genetic variations linked to different diseases and environmental factors like smoking and air pollution, Manuguerra et al. [75] used MDR on patients with myeloid leukaemia, bladder cancer, and lung cancer. With a prediction error of 0.74 and a CVC of 6.60, the optimal interaction model between the environmental variable and the investigated genes, XRCC1_28152 and BRCA2, was found for lung cancer. The four-locus models (APE1, RAD52, COMT, and MTHFR) for bladder cancer provided a prediction error of 0.78 and a CVC of 6.60 and 0.78 with a CVC of 7.40. MDR was used in case–control studies of breast cancer in Finnish and Spanish populations by Milne et al. [76]. With a test accuracy ranging from 56 to 58%, MDR revealed a quadruple interaction between the SNPs rs40419, rs2267922, rs2498804, and rs93059 in the B-cell receptor pathway. Mostowska et al. [77] used MDR on DNMT1, DNMT3A, and DNMT3B genes to determine the risk of ovarian cancer via genetic interactions. With a balanced accuracy of 59.03%, MDR determined that the optimal SNPs combinations were rs759920, rs2289195, rs7590760, and rs2424932. Moreover, MDR was applied by Marcus et al. [78] to predict lung cancer by relying on epistatic interactions. Three SNPs: rs1799732, rs5744256, and rs2306022 from DRD2, IL-18, and ITGA11 were among the five SNPs that MDR selected as best associated with lung cancer.

The major strength of the current study was the application of the risk-prediction model: MDR. The limitations of this study should be noted: (i) This work was carried out in a single recruitment centre. (ii) The number of subjects included was limited to the possibilities of recruitment in a single geographical area. Representative studies of larger and more diverse populations are needed if precision medicine and prevention are to be applied to the whole population. (iii) Our study did not include subjects who had undergone prostatectomy. (iv) Furthermore, assessing acetylated ghrelin would help in the investigation of potential relationships.

## 5. Conclusions

Here, we highlight the non-association of the *GHRL* (rs696217, rs4684677, rs3491141) and *GHSR* (rs2922126, rs572169, rs2948694) variant gene polymorphisms with PCa in Algerian patients. Furthermore, our results also suggest that the TT genotype of the rs2922126 SNP is associated with sporadic PCa and could be a PCa susceptibility genotype. The effect of the gene–gene interaction between the rs2922126 and rs572169 polymorphisms appears to play an important role in the pathogenesis of PCa. In fact, while each of these SNPs has no effect by itself, a combined model promotes the risk of PCa. Therefore, MDR epistasis analysis may be a useful technique for identifying people who are at high risk of developing PCa and may be used as a therapeutic target.

We cannot conclude from an association study whether the measured allele is functionally responsible for the effect. Further examination of the pleiotropic effect of the alleles can provide clues; however, *in vitro* and *in vivo* functional analyses are required to concretely establish the effect of the genotyped alleles. Moreover, study on the epigenetic mechanism underlying PCa and the *GHRL* and *GHSR* genes may be relevant to provide concise information about the impact of the allelic variants.

## Figures and Tables

**Table 1:** Demographic and clinical characteristics of the study groups.

Characteristics	Controls	Patients	*p*-Value
**Age (years)**			
Mean ± SD	71.59 ± 8.476	74.63 ± 9.50	0.015
**BMI (kg/m^2^)**			
Mean ± SD	22.89 ± 1.304	22.29 ± 2.034	0.013
**Residence**			
Urban	76	81	
Rural	19	14	
**PSA (ng/mL)**			
Mean ± SD	4.26 ± 3.48	80.72 ± 24.34	0.001
**Gleason Score**			
<7 (Low)	-	38 (31.66)	
7 (Intermediate)	-	48 (40.00)	NA
>7 (High)	-	34 (28.33)	
**Clinical Stade**			
Localised	-	70 (58.33)	
Advanced	-	34 (28.33)	NA
Metastatic	-	16 (13.33)	
**Death**	-	19 (15.43)	NA

NA: not applicable.

**Table 2:** Characteristics of the studied SNPs.

Gene	SNP	Position	Allele	MAF	Localization	SNP Type	TaqMan SNP
***GHRL*** **3p25.3**	rs4684677	10286769	T > A	0.060	Exon 4	Missense (Gln 90 Leu)	C__25607748_10
	rs696217	10289773	G > T	0.077	Exon 3	Missense (Leu 72 Met)	C___3151003_20
	rs34911341	10289835	C > T	0.007	Exon 3	Missense (Arg 51 Gln)	C__25607739_20
***GHSR*** **3q26.31**	rs2948694	172447373	T/C, G, T	0.110	Intron 1	Intron	C__16174361_10
	rs572169	172447937	C > T	0.295	Exon 1	Silent	C___1079489_20
	rs2922126	172449471	T > A	0.303	2KB upstream variant	5′ flanking region/promoter	C___3261006_10

SNP: single nucleotide polymorphism; *GHRL*: pro-ghrelin gene; *GHSR*: growth hormone secretagogue receptor gene.

**Table 3:** HWE for *GHRL* (rs696217, rs4684677, and rs34911341) and *GHSR* (rs2922126, rs572169, and rs2948694).

Gene	SNPs	HWE	No-HWE	χ^2^	MAF
***GHRL***	rs696217	-	*p* = 2 × 10^−4^	8.021	0.199
	rs4684677	*p* = 1.000	-	0.002	0.005
	rs34911341	*p* = 1.000	-	-	0.000
***GHSR***	rs2922126	-	*p* = 5.106 × 10^−4^	15.336	0.353
	rs572169	-	*p* = 2.876 × 10^−7^	17.941	0.275
	rs2948694	*p* = 1.000	-	0.183	0.032

If *p* < 0.05, the SNP is not in HWE.

**Table 4:** Allelic distribution at *GHRL* (rs4684677, rs696217, and rs34911341) and *GHSR* (rs2922126, rs572169, and rs2948694) gene loci in PCa patients and healthy controls.

Gene	Locus	Allele	Controls	Patients	OR (95% CI)	*p*-Value
***GHRL***	rs34911341					
	C > T	C	170 (1.000)	200 (1.000)	-	-
	rs696217		n = 168	n = 214		
	G > T	G	138 (0.821)	168 (0.785)	0.794 (0.475~1.325)	0.376
		T	30 (0.178)	46 (0.215)		
	rs4684677		n = 172	n = 208		
	A > G	A	171 (0.994)	207 (0.995)	1.210 (0.075~19.497)	0.892
		G	1 (0.005)	1 (0.004)		
***GHSR***						
	rs2922126		n = 174	n = 240		
	T > A	A	118 (0.678)	150 (0.625)	0.791 (0.524~1.193)	0.263
		T	56 (0.321)	90 (0.375)		
	rs572169		n = 160	n = 204		
	C > T	C	118 (0.738)	147 (0.721)	0.918 (0.575~1.463)	0.718
		T	42 (0.263)	57 (0.263)		
	rs2948694		n = 170	n = 238		
	T/C, G, A	A	167 (0.982)	229 (0.962)	2.188 (0.121~1.714)	0.234
		G	3 (0.017)	9 (0.037)		

**Table 5:** Genotypic distribution at *GHRL* (rs4684677, rs696217, and rs34911341) and *GHSR* (rs2922126, rs572169, and rs2948694) gene loci in PCa patients and healthy controls.

Gene	Locus	Genotype	Controls	Patients	OR (95% CI)	*p*-Value
***GHRL***	rs34911341	C/C	85 (1.000)	100 (1.000)	-	*-*
	rs696217	G/G	54 (0.642)	61 (0.570)	0.736 (0.409–1.326)	0.307
		G/T	30 (0.357)	46 (0.429)		
	rs4684677	A/A	85 (0.988)	103 (0.990)	1.211 (0.074~19.663)	0.892
		A/G	1 (0.011)	1 (0.009)		
***GHSR***	rs2922126	A/A	31 (0.356)	36 (0.300)	1.292 (0.714–2.317)	0.452
		A/T	56 (0.644)	78 (0.650)	0.972 (0.555–1.729)	0.999
		T/T	0 (0.000)	6 (0.050)	-	0.040
	rs572169	C/C	38 (0.475)	45 (0.441)	0.872 (0.484–1.570)	0.649
		C/T	42 (0.525)	57 (0.559)		
			82 (0.965)			
	rs2948694	A/A	3 (0.035)	110 (0.924)	0.447 (0.117–1.7703)	0.227
		A/G		9 (0.076)		

**Table 6:** Haplotype analysis of SNPs in the *GHRL* and *GHSR* genes.

Gene	Haplotype ^#^	Frequencies	Controls	Patients	χ^2^	*p*-Value
***GHRL***	AGC	0.798	0.815	0.784	0.574	0.448
	ATC	0.197	0.179	0.211	0.621	0.430
***GHSR***	ACA	0.587	0.599	0.577	0.195	0.658
	ATT	0.211	0.191	0.226	0.747	0.387
	ACT	0.121	0.122	0.120	0.002	0.968
	ATA	0.048	0.065	0.036	1.757	0.184
	GTA	0.018	0.016	0.019	0.061	0.805
	GCT	0.015	0.007	0.020	1.156	0.282

**#** Based on physical order of markers: *GHRL* (rs34911341, rs696217, and rs4684677) and *GHSR* (rs2922126, rs572169, and rs2948694).

**Table 7:** Results of MDR analysis.

Modèles	Training Balanced Accuracy	Testing Balanced Accuracy	CVC	F-Value	MCC
rs2922126	0.6780	0.5631	7/10	-	-
rs572169, rs2922126	0.8235	0.8108	10/10	0.953	1.544
rs572169, rs696217, rs2922126	0.8335	0.8198	7/10	-	-
rs572169, rs34911341, rs696217, rs2922126	0.8370	0.8198	7/10	-	-
rs572169, rs34911341, rs696217, rs4684677, rs2922126	0.8003	0.6622	10/10	-	-

CVC: cross-validation consistency; MCC: Matthews correlation coefficient.

## Data Availability

The data that support the findings of this study are available from the corresponding authors upon reasonable request.

## References

[1] GLOBOCAN 2020. New Global Cancer Data. www.uicc.org/news/globocan-2020-new-global-cancer-data.

[2] Bray F., Ferlay J., Soerjomataram I., Siegel R.L., Torre L.A., Jemal A. (2018). Global cancer statistics 2018: GLOBOCAN estimates of incidence and mortality worldwide for 36 cancers in 185 countries. Cancer J. Clin..

[3] Feigelson H.S., Goddard K.A., Hollombe C., Tingle S.R., Gillanders E.M., Mechanic L.E., Nelson S.A. (2014). Approaches to integrating germline and tumor genomic data in cancer research. Carcinogenesis.

[4] Hicks C., Miele L., Koganti T., Vijayakumar S. (2013). Comprehensive assessment and network analysis of the emerging genetic susceptibility landscape of prostate cancer. Cancer Inform..

[5] Bourefis A., Berredjem H., Djeffal O., Le T.K., Giusiano S., Rocchi P. (2022). HSP27/Menin Expression as New Prognostic Serum Biomarkers of Prostate Cancer Aggressiveness Independent of PSA. Cancers.

[6] Bukhari S.N.A. (2022). An insight into the multifunctional role of ghrelin and structure activity relationship studies of ghrelin receptor ligands with clinical trials. Eur. J. Med. Chem..

[7] Kojima M., Hosoda H., Date Y., Nakazato M., Matsuo H., Kangawa K. (1999). Ghrelin is a growth-hormone-releasing acylated peptide from stomach. Nature.

[8] Asakawa A., Inui A., Kaga T., Yuzuriha H., Nagata T., Ueno N., Makino S., Fujimiya M., Niijima A., Fujino M.A. (2001). Ghrelin is an appetite-stimulatory signal from stomach with structural resemblance to motilin. Gastroenterology.

[9] Gahete M.D., Rincon-Fernandez D., Villa-Osaba A., Hormaechea-Agulla D., Ibanez-Costa A., Martinez-Fuentes A.J., Gracia-Navarro F., Castaño J.P., Luque R.M. (2014). Ghrelin gene products, receptors, and GOAT enzyme: Biological and pathophysiological insight. J. Endocrinol..

[10] Muller T.D., Nogueiras R., Andermann M.L., Andrews Z.B., Anker S.D., Argente J., Batterham R.L., Benoit S.C., Bowers C.Y., Broglio T. (2015). Ghrelin. Mol. Metab..

[11] Lanfranco F., Bonelli L., Baldi M., Me E., Broglio F., Ghigo E. (2008). Acylated ghrelin inhibits spontaneous luteinizing hormone pulsatility and responsiveness to naloxone but not that to gonadotropin-releasing hormone in young men: Evidence for a central inhibitory action of ghrelin on the gonadal axis. J. Clin. Endocrinol. Metab..

[12] Kotta A.S., Kelling A.S., Corleto K.A., Sun Y., Giles E.D. (2022). Ghrelin and Cancer: Examining the Roles of the Ghrelin Axis in Tumor Growth and Progression. Biomolecules.

[13] Lin T.C., Liu Y.P., Chan Y.C., Su C.Y., Lin Y.F., Hsu S.L., Yang C.S., Hsiao M. (2015). Ghrelin promotes renal cell carcinoma metastasis via Snail activation and is associated with poor prognosis. J. Pathol..

[14] Kraus D., Reckenbeil J., Wenghoefer M., Stark H., Frentzen M., Allam J.P., Novak N., Frede S., Götz W., Probstmeier R. (2016). Ghrelin promotes oral tumor cell proliferation by modifying GLUT1 expression. Cell. Mol. Life Sci..

[15] Villarreal D., Pradhan G., Zhou Y., Xue B., Sun Y. (2022). Diverse and Complementary Effects of Ghrelin and Obestatin. Biomolecules.

[16] Duxbury M.S., Waseem T., Ito H., Robinson M.K., Zinner M.J., Ashley S.W., Whang E.E. (2003). Ghrelin promotes pancreatic adenocarcinoma cellular proliferation and invasiveness. Biochem. Biophys. Res. Commun..

[17] Yeh A.H., Jeffery P.L., Duncan R.P., Herington A.C., Chopin L.K. (2005). Ghrelin and a novel preproghrelin isoform are highly expressed in prostate cancer and ghrelin activates mitogen-activated protein kinase in prostate cancer. Clin. Cancer Res..

[18] Spiridon I.A., Apostol Ciobanu D.G., Giușcă S.E., Căruntu D.I. (2021). Ghrelin and its role in gastrointestinal tract tumors (Review). Mol. Med. Rev..

[19] Cassoni P., Ghé C., Marrocco T., Tarabra E., Allia E., Catapano F., Deghenghi R., Ghigo E., Papotti M., Muccioli G. (2004). Expression of ghrelin and biological activity of specific receptors for ghrelin and des-acyl ghrelin in human prostate neoplasms and related cell lines. Eur. J. Endocrinol..

[20] Jin Y., Borell H., Gardin A., Ufer M., Huth F., Camenisch G. (2018). In vitro studies and in silico predictions of fluconazole and CYP2C9 genetic polymorphism impact on siponimod metabolism and pharmacokinetics. Eur. J. Clin. Pharmacol..

[21] Garcia E.A., King P., Sidhu K., Ohgusu H., Walley A., Lecoeur C., Gueorguiev M., Khalaf S., Davies D., Grossman A.B. (2009). The role of ghrelin and ghrelin-receptor gene variants and promoter activity in type 2 diabetes. Eur. J. Endocrinol..

[22] Liao N., Xie Z.K., Huang J., Xie Z.F. (2013). Association between the ghrelin Leu72Met polymorphism and type 2 diabetes risk: A meta-analysis. Gene.

[23] Gjesing A.P., Larsen L.H., Torekov S.S., Hainerova I.A., Kapur R., Johansen A., Albrechtsen A., Boj S., Holst B., Harper A. (2010). Family and population-based studies of variation within the ghrelin receptor locus in relation to measures of obesity. PLoS ONE.

[24] Ukkola O., Paakko T., Kesaniemi Y.A. (2011). Ghrelin and its promoter variant associated with cardiac hypertrophy. J. Hum. Hypertens..

[25] Mora M., Adam V., Palomera E., Blesa S., Díaz G., Buquet X., Serra-Prat M., Martín-Escudero J.C., Palanca A., Chaves J.F. (2015). Ghrelin Gene Variants Influence on Metabolic Syndrome Components in Aged Spanish Population. PLoS ONE.

[26] Berthold H.K., Giannakidou E., Krone W., Tregouet D.A., Gouni-Berthold I. (2010). Influence of ghrelin gene polymorphisms on hypertension and atherosclerotic disease. Hypertens. Res..

[27] Hamdy M., Kassim S.K., Khairy E., Maher M., Mansour K.A., Albreedy A.M. (2018). Ghrelin gene polymorphism as a genetic biomarker for prediction of therapy induced clearance in Egyptian chronic HCV patients. Gene.

[28] Dardennes R.M., Zizzari P., Tolle V., Foulon C., Kipma A., Romo L., Iancu-Gontard D., Boni C., Sinet P.M., Bluet M.T. (2007). Family trios analysis of common polymorphisms in the obestatin/ghrelin, BDNF and AGRP genes in patients with Anorexia nervosa: Association with subtype, body-mass index, severity and age of onset. Psychoneuroendocrinology.

[29] Blum D., Wolf-Linder S., Oberholzer R., Brändle M., Hundsberger T., Strasser F. (2021). Natural ghrelin in advanced cancer patients with cachexia, a case series. J. Cachexia Sarcopenia Muscle.

[30] Landgren S., Jerlhag E., Zetterberg H., Gonzalez-Quintela A., Campos J., Olofsson U., Nilsson S., Blennow K., Engel J.A. (2008). Association of pro-ghrelin and GHS-R1A gene polymorphisms and haplotypes with heavy alcohol use and body mass. Alcohol Clin. Exp. Res..

[31] Langre S., Berglund K., Jerlhag E., Fahlke C., Balldin J., Berggren U., Zetterberg H., Blennow K., Jörgen A. (2011). Reward-Related Genes and Personality Traits in Alcohol-Dependent Individuals: A Pilot Case Control Study. Neuropsychobiology.

[32] Steinle N.I., Pollin T.I., O’Connell J.R., Mitchell B.D., Shuldiner A.R. (2005). Variants in the ghrelin gene are associated with metabolic syndrome in the Old Order Amish. J. Clin. Endocrinol. Metab..

[33] Hansson C., Annerbrink K., Nilsson S., Bah J., Olsson M., Allgulander C., Andersch S., Sjodin I., Eriksson E., Dickson S.L. (2013). A possible association between panic disorder and a polymorphism in the preproghrelingene. Psychiatry Res..

[34] Pabalan N.A., Seim I., Jarjanazi H., Chopin L.K. (2014). Associations between ghrelin and ghrelin receptor polymorphisms and cancer in Caucasian populations: A meta-analysis. BMC Genet..

[35] Moniuszko A., Wawrusiewicz-Kurylonek N., Bossowska A., Goscik J., Luczynski W., Glowinska-Olszewska B., Kretowski A., Bossowski A. (2015). The association between rs4684677 T/A polymorphism in preproghrelin gene and predisposition to autoimmune thyroid diseases in children. Autoimmunity.

[36] Doecke J.D., Zhao Z.Z., Stark M.S., Green A.C., Hayward N.K., Montgomery G.W., Webb P.M., Whiteman D.C. (2008). Single nucleotide polymorphisms in obesity related genes and the risk of esophageal cancers. Cancer Epidemiol. Biomark. Prev..

[37] Campa D., Pardini B., Naccarati A., Vodickova L., Novotny J., Steinke V., Rahner N., Holinski-Feder E., Morak M., Schackert H.K. (2010). Polymorphisms of genes coding for ghrelin and its receptor in relation to colorectal cancer risk: A two-step gene-wide case-control study. Gastroenterology.

[38] Kasprzak A. (2022). Role of the Ghrelin System in Colorectal Cancer. Int. J. Mol. Sci..

[39] Dossus L., McKay J.D., Canzian F., Wilkening S., Rinaldi S., Biessy C., Olsen A., Tjønneland A., Jakobsen M.U., Overvad K. (2008). Polymorphisms of genes coding for ghrelin and its receptor in relation to anthropometry, circulating levels of IGF-I and IGFBP-3, and breast cancer risk: A case–control study nested within the European Prospective Investigation into Cancer and Nutrition (EPIC). Carcinogenesis.

[40] Skibola D.R., Smith M.T., Bracci P.M., Hubbard A.E., Agana L., Chi S., Holly E.A. (2005). Polymorphisms in ghrelin and neuropeptide Y genes are associated with non-Hodgkin lymphoma. Cancer Epidemiol. Biomark. Prev..

[41] Chopin L.K., Seim I., Walpole C.M., Herington A.C. (2012). The ghrelin axis: Does it have an appetite for cancer progression?. Endocr. Rev..

[42] Han Q., Shan Z., Hu J., Zhang N. (2015). Relationship between gene polymorphisms and prostate cancer risk. Asian Pac. J. Trop. Med..

[43] Hormaechea-Agulla D., Gahete M.D., Jiménez-Vacas J.M., Gómez-Gómez E., Ibáñez-Costa A., López F.L., Rivero-Cortés E., Sarmento-Cabral A., Valero-Rosa J., Carrasco-Valiente J. (2017). The oncogenic role of the In1-ghrelin splicing variant in prostate cancer aggressiveness. Mol. Cancer.

[44] Jiménez-Vacas J.M., Montero-Hidalgo A.J., Gómez-Gómez E., Fuentes-Fayos A.C., Ruiz-Pino F., Guler I., Camargo A., Anglada F.J., Carrasco-Valiente J., Tena-Sempere M. (2021). In1-Ghrelin Splicing Variant as a Key Element in the Pathophysiological Association Between Obesity and Prostate Cancer. J. Clin. Endocrinol. Metab..

[45] Barrett J.C., Fry B., Maller J., Daly M.J. (2005). Haploview: Analysis and visualization of LD and haplotype maps. Bioinformatics.

[46] Ritchie M.D., Hahn L.W., Roodi N., Bailey R., Dupont W.D., Parl F.F., Moore J.H. (2001). Multifactor-Dimensionality Reduction Reveals High-Order Interactions among Estrogen Metabolism Genes in Sporadic Breast Cancer. Am. J. Hum. Genet..

[47] Hahn L.W., Ritchie M.D., Moore J.H. (2003). Multifactor dimensionality reduction software for detecting gene-gene and geneenvironment interactions. Bioinformatics.

[48] Moore J.H., Gilbert J.C., Tsai C.T., Chiang F.T., Holden T.B.N., White B.C. (2006). A flexible computational framework for detecting, characterizing, and interpreting statistical patterns of epistasis in genetic studies of human disease susceptibility. J. Theor. Biol..

[49] Freeman V.L., Meydani M., Hurr K., Flanigan R.C. (2004). Inverse association between prostatic polyunsaturated fatty acid and risk of locally advanced prostate carcinoma. Cancer.

[50] Gallagher E.J., LeRoith D. (2011). Diabetes, cancer, and metformin: Connections of metabolism and cell proliferation. Ann. N. Y. Acad. Sci..

[51] Travis R.C., Appleby P.N., Martin R.M., Holly J.M., Albanes D., Black A., Bueno-de-Mesquita H.B., Chan J.M., Chen C., Chirlaque M.D. (2016). A meta-analysis of individual participant data reveals an association between circulating levels of IGF-I and prostate cancer risk. Cancer Res..

[52] Zhang X., Zhai L., Rong C., Qin X., Shan L. (2005). Association of Ghrelin Gene Polymorphisms and Serum Ghrelin Levels with the Risk of Hepatitis B Virus-Related Liver Diseases in a Chinese Population. PLoS ONE.

[53] Wang W., Chen Z.X., Guo D.Y., Tao Y.X. (2018). Regulation of prostate cancer by hormone-responsive G protein-coupled receptors. Pharmacol. Ther..

[54] Zhang Q., Huang W.D., Lv X.Y., Yang Y.M. (2011). Ghrelin protects H9c2 cells from hydrogen peroxide-induced apoptosis through NF-kappaB and mitochondria-mediated signaling. Eur. J. Pharmacol..

[55] Liu B., Garcia E.A., Korbonits M. (2011). Genetic studies on the ghrelin, growth hormone secretagogue receptor (GHSR) and ghrelin O-acyl transferase (GOAT) genes. Peptides.

[56] Marzolini C., Paus E., Buclin T., Kim R.B. (2004). Polymorphisms in human MDR1 (Pglycoprotein): Recent advances and clinical relevance. Clin. Pharmacol. Ther..

[57] Espigolan R., Baldi F., Boligon A.A., Souza F.R., Gordo D.G., Tonussi R.L., Cardoso D.F., Oliveira H.N., Tonhati H., Sargolzaei M. (2013). Study of whole genome linkage disequilibrium in Nellore cattle. BMC Genom..

[58] Porto-Neto L.R., Kijas J.W., Reverter A. (2014). The extent of linkage disequilibrium in beef cattle breeds using high-density SNP genotypes. Genet. Sel. Evol..

[59] Fleck J.L., Pavel A.B., Cassandras C.G. (2016). Integrating mutation and gene expression cross-sectional data to infer cancer progression. BMC Syst. Biol..

[60] Jia P., Zhao Z. (2017). Impacts of somatic mutations on gene expression: An association perspective. Brief. Bioinform..

[61] Lin T.C., Hsiao M. (2017). Ghrelin and cancer progression. Biochim. Biophys. Acta BBA Rev. Cancer.

[62] Lanktree M.B., Guo Y., Murtaza M., Joseph T., Glessner J.T., Bailey S.D., Onland-Moret N.C., Lettre G., Ongen H., Rajagopalan R. (2011). Meta-analysis of Dense Genecentric Association Studies Reveals Common and Uncommon Variants Associated with Height. Am. J. Hum. Genet..

[63] Fernández Torres J., Martínez Nava G.A., Zamudio Cuevas Y., Martínez Flores K., Espinosa Morales R. (2019). Epistasis between ADIPOQ rs1501299 and PON1 rs662 polymorphisms is potentially associated with the development of knee osteoarthritis. Mol. Biol. Rep..

[64] Manavalan R., Priya S. (2021). Genetic interactions effects for cancer disease identification using computational models: A review. Med. Biol. Eng. Comput..

[65] Moore J.H., Williams S.M. (2002). New Strategies for Identifying Gene-gene Interactions in Hypertension. Ann. Med..

[66] Nagel R.I. (2002). Epistasis and the Genetics of Human Diseases. Comptes Rendus Biol..

[67] Kardia S.L.R. (2000). Context-Dependent Genetic Effects in Hypertension. Curr. Hypertens. Rep..

[68] Templeton A.R., Wade M., Brodie B., Wolf J. (2000). Epistasis and Complex Traits. Epistasis and the Evolutionary Process.

[69] Herbert A., Gerry N.P., McQueen M.B., Heid I.M., Pfeufer A., Illig T., Wichmann H.E., Meitinger T., Hunter D., Hu F.B. (2006). A Common Genetic Variant is Associated with Adult and Childhood Obesity. J. Comput. Biol..

[70] Lambert J.C., Heath S., Even G., Campion D., Sleegers K., Hiltunen M., Combarros O., Zelenika D., Bullido M.J., Tavernier B. (2009). Genome-Wide Association Study Identifies Variants at CLU and CR1 Associated with Alzheimer’s disease. Nat. Genet..

[71] Marchini J., Donnelley P., Cardon L.R. (2005). Genome-Wide Strategies for Detecting Multiple Loci that Influence Complex Diseases. Nat. Genet..

[72] Velez D.R., White B.C., Motsinger A.A., Bush W.S., Ritchie M.D., Williams S.M., Moore J.H. (2007). A Balanced Accuracy Function for Epistasis Modeling in Imbalanced Dataset using Multifactor Dimensionality Reduction. Genet. Epidemiol..

[73] Jiang X., Barmada M., Visweswaran S. (2010). Identifying Genetic Interactions in Genome-Wide Data Using Bayesian Networks. Genet. Epidemiol..

[74] Moore J.H., Williams S.M. (2009). Epistasis and its implications for personal genetics. Am. J. Hum. Genet..

[75] Manuguerra M., Matullo G., Veglia F., Autrup H., Dunning A.M., Garte S., Gormally E., Malaveille C., Guarrera S., Polidoro S. (2007). Multi-factor dimensionality reduction applied to a large prospective investigation on gene-gene and gene-environment interactions. Carcinogenesis.

[76] Milne R.I., Fagerholm R., Nevanlinna H., BenÍtez J. (2008). The importance of replication in gene-gene interaction studies: Multifactor dimensionality reduction applied to a two-stage breast cancer case-control study. Carcinogenesis.

[77] Mostowska A., Sajdak S., Pawlik P., Lianeri M., Jagodzinski P.P. (2013). DNMT1, DNMT3A and DNMT3B gene variants in relation to ovarian cancer risk in the Polish population. Mol. Biol. Rep..

[78] Marcus M.W., Raji O.Y., Duffy S.W., Young R.P., Hopkins R.J., Field J.K. (2016). Incorporating epistasis interaction of genetic susceptibility single nucleotide polymorphisms in a lung cancer risk prediction model. Int. J. Oncol..

